# Street Dust—Bound Polycyclic Aromatic Hydrocarbons in a Saudi Coastal City: Status, Profile, Sources, and Human Health Risk Assessment

**DOI:** 10.3390/ijerph15112397

**Published:** 2018-10-29

**Authors:** Ibrahim I. Shabbaj, Mansour A. Alghamdi, Mamdouh I. Khoder

**Affiliations:** Department of Environmental Sciences, Faculty of Meteorology, Environment and Arid Land Agriculture, King Abdulaziz University, P.O. Box 80208, Jeddah 21589, Saudi Arabia; ishabbaj@kau.edu.sa (I.I.S.); mkhader@kau.edu.sa (M.I.K.)

**Keywords:** street dust, PAHs, source evaluation, incremental lifetime cancer risk, cancer risk assessment, coastal city

## Abstract

Polycyclic aromatic hydrocarbons (PAHs) in street dust pose a serious problem threatening both the environment and human health. Street dust samples were collected from five different land use patterns (traffic areas TRA, urban area URA, residential areas REA, mixed residential commercial areas MCRA and suburban areas SUA) in Jeddah, a Saudi coastal city, and one in in Hada Al Sham, a rural area (RUA). This study aimed to investigate the status, profile, sources of PAHs and estimate their human health risk. The results revealed an average concentration of total PAHs of 3320 ng/g in street dust of Jeddah and 223 ng/g in RUA dust. PAHs with high molecular weight represented 83.38% of total PAHs in street dust of Jeddah, while the carcinogenic PAH compounds accounted 57.84%. The highest average concentration of total PAHs in street dust of Jeddah was found in TRA (4980 ng/g) and the lowest in REA (1660 ng/g). PAHs ratios indicated that the principal source of PAHs in street dust of Jeddah is pyrogenic, mainly traffic emission. Benzo(a)anthracene/chrysene (BaA/CHR) ratio suggests that PAHs in street dusts of Jeddah come mainly from emission of local sources, while PAHs in RUA might be transported from the surrounding urban areas. The estimated Incremental Lifetime Cancer Risk (ILCR) associated with exposure to PAHs in street dusts indicated that both dermal contact and ingestion pathways are major contributed to cancer risk for both children and adults. Based on BaP_equivalence_ concentrations of total PAHs, ILCR_Ingestion_, ILCR_dermal_ and cancer risk values for children and adults exposed to PAHs in street dust of different areas in Jeddah were found between 10^−6^ and 10^−4^, indicating potential risk. The sequence of cancer risk was TRA > URA > MCRA > SUA > REA. Only exposure to BaP and DBA compounds had potential risk for both children and adults.

## 1. Introduction

Surface street dust particles are considered to be one of the most important sources of fine aerosols in urban atmospheres and can become easily airborne through wind dispersion [1,2,3]. Atmospheric aerosols and their contaminants from anthropogenic sources finally settle on the surfaces by atmospheric dry and/or wet deposition, and are then transferred to the surface of the soil or incorporated into the surface dusts. The chemical contents of the surface road dust is related to atmospheric particulate content and their chemical composition in both road dust and airborne particulate are similar [4]. Vehicle exhaust, tire dust, spillages and leaks from vehicles, road surface erosion material and vegetative plant fragments, garden soil, and litter are the sources of deposited surface roadside dust [5,6,7]. Road dust is a non-point source of polycyclic aromatic hydrocarbons (PAHs) [4,8]. The levels of PAHs in sediment, marine, water, and food are affected by the presence of PAH in street dust [7]. Direct inhalation of fine dust by people traversing the streets and those residing in the vicinity, ingestion through hand-to-mouth, eating poorly washed fruits and vegetables, and dermal exposure are the routes of human exposure to road dust [9,10,11]. Chemical composition of road dust can be used as an indicator for environmental pollution [12], a valuable medium for characterizing urban environmental quality [13], and exposure health risk assessment [8,14].

PAHs are long-lived and ubiquitous air pollutants in the environment [15,16,17]. They are composed of two or more fused aromatic rings [18]. They arise mainly from incomplete combustion processes. The emission sources of PAHs include road traffic, domestic combustion, power plants, industrial combustion, cigarette smoke, petroleum refining, coal, straw and firewood burning, oil spills and coal tars, wood burning, fireplaces, charcoal-grilled, smoked food, steel plants and the petrochemical and metallurgical industries [19,20,21,22,23]. PAHs sources may be pyrogenic and/or petrogenic. Pyrogenic sources are fossil fuel combustion and traffic exhaust, whereas petrogenic sources are crude oil and petroleum products [2]. Tire abrasion and tailpipe discharge, coal combustion products, industrial emissions, crank case oil, oil combustion, wood emission, open waste burning and, asphalt and tyre rubber are the major sources of pyrogenic and petrogenic PAHs [4,6,7,24]. Dong and Lee [6] indicated that the traffic density and cleaning frequency affect the levels of PAH in road dust. They also found high levels of PAH in lower vehicular traffic areas, indicating that PAHs in road dust are affected by other sources.

Although many studies measured the levels of PAHs in road dust [4,6,25,26], their health risk is unclear especially in urbanized areas. Krugly et al. [15] and Wang et al. [11] revealed that road dust contaminated by PAH leads to increase the health risk of children and adults. PAHs are of special interest in the context of public health as some congeners are highly carcinogenic, and the PAH mixture has been classified by IARC as a proven human carcinogen [27]. Epidemiologic studies revealed increased lung, skin, and bladder cancer risks for employees exposed to PAHs [28,29,30,31,32], as well as for residents of urban and nearby neighborhoods [33,34,35]. Inhalation of PAHs cause allergic responses and impaired fetal development [36,37,38].

In Saudi Arabia, the coastal city of Jeddah, with a population of ca. 3.4 million, is considered to be the most significant commercial centre. Due to rapid and diverse growth, Jeddah shows an environmental deterioration. Increased number of vehicles led to an increased rate of air quality deterioration. Power plant, desalination plant and refinery are the biggest stationary PAH sources in Jeddah city. Although there are several studies regarding the levels of PAHs in street dust in the world [8,39,40,41,42,43,44], information about the levels, distributions, sources and health risk assessment of PAHs in surface street dust of Jeddah is scarce. This poses difficulties on air pollution control and management. Previous studies on air pollution in Jeddah focused on pollutants like NO_2_, O_3_, BTEX, PM_10_, PM_2.5_ and their elemental composition, PAHs in gaseous and total suspended particulate phases and only heavy metals in street dust [45,46,47,48,49,50,51,52,53,54]. Therefore, the main objectives of the present study were as follows: (1) to evaluate the PAHs concentration levels content in the surface street dust which collected from different land use patterns in Jeddah and Hada Al Sham, (2) to characterize the distribution and profiles of each PAH compound in the collected surface street dust, (3) to identify the PAHs sources, using diagnostic ratios of PAH compounds in the collected surface street dust, and (4) to assess the human health risks due to exposure of PAHs in the surface street dusts in children and adults via ingestion, inhalation and dermal contact.

## 2. Materials and Methods

### 2.1. Sampling Sites Description

**Figure 1:**
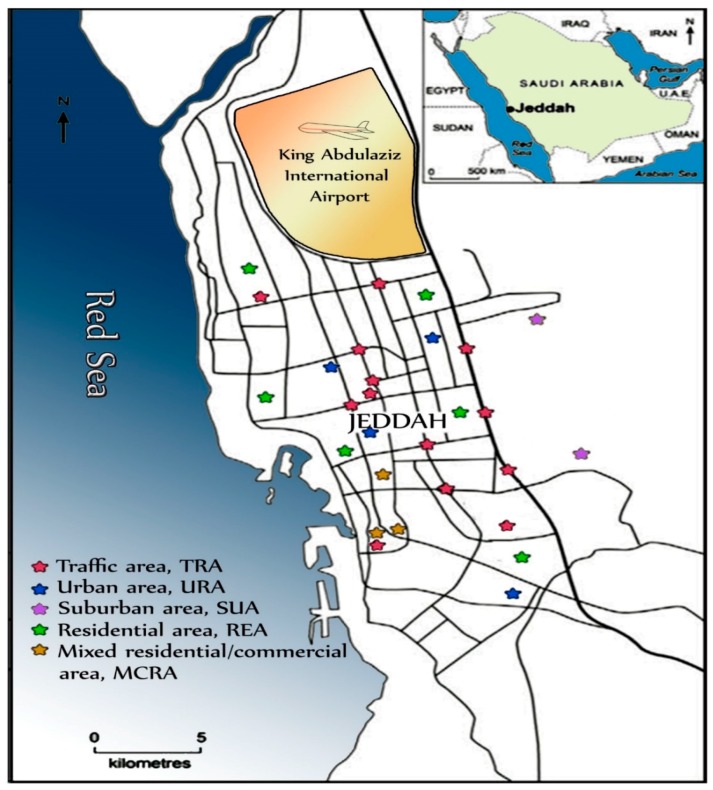
Sampling site distribution in the different land use patterns in Jeddah.

Surface street dust samples were collected from five different land use patterns in Jeddah viz; traffic area (TRA, major highway, roundabouts, crossroads and parks), residential area (REA), mixed commercial/residential area (MCRA, residential communities and commercial activities), urban area (URA) and suburban area (SUA) (Figure 1). These areas were selected to represent varied functional categories and to detect the impact of pollution from different activities. Surface street dust samples were collected also from a rural area (RUA), located at Hada Al Sham, about 60 km east of the city of Jeddah.

### 2.2. Sample Collection

Gentle sweeping motions using plastic brushes and dustpans were used to collect the surface street dust samples, especially fine dust [54]. A total of 87 surface street dust samples were collected from five different land use patterns in Jeddah and a rural area in Hada Al Sham. Street dust samples were collected during September 2017 on weekdays. Approximately 300 gm of surface street dust on impenetrable surfaces of the paved roads among 8-m radius circle were collected from each land use type. Brushes and dustpans were cleaned after each sampling. Bags, wrapped with solvent (*n*-hexane)-rinsed aluminum foil, were used to store the collected street dust samples. They were sealed in polyethylene bags and transported to the lab. The samples were set in a desiccator to remove wetness. The coarse impurities of the samples such as cigarette buts, small gravel, plastic waste, metal scraps, and broken construction debris were removed using 1.0 mm mesh nylon sieves. Then, the remaining part of the sample was homogenized, sieved through a <63 µm particle size sieve and stored in small self-sealing plastic bags in a refrigerator until analysis.

### 2.3. Extraction of PAHs

A known weight (2 gm) of the surface street dust samples were Soxhlet extracted for 16 h [55]. A mixture of acetone/dichloromethane/*n*-hexane (1:1:1, *v*/*v*/*v*) was used for extraction. The extracted organic part was concentrated using a rotary evaporator, and cleaned-up according to Park et al. [56]. The collected eluent from the clean-up procedure was concentrated and exchanged to 1 ml hexane and stored frozen (−10 °C) until analysis.

### 2.4. Analysis of PAHs

Gas chromatography (GC)/flame ionization detector (FID) was used for determination the concentration of PAH compounds, using PAH standards. One microliter (μL) of extract was withdrawn from the samples, including blanks, and injected into a Hewlett-Packard (HP6890, Agilent, Santa Clara, CA, USA) gas chromatograph (GC), fitted with a flame ionization detector (FID). A HP-5 capillary column was used with hydrogen as carrier gas. The concentrations of the target PAH compounds were quantified by an external standard solution of 15 PAH compounds (PAH mixture, Supelco, Inc., St. Louis, MO, USA). To check the reliability of the obtained results, Quality assurance/quality control (QA/QC) including reagent blanks, analytical standards, standard spike recoveries, GC/FID calibration and detection limits were employed. Based on the drift in retention times and responses of PAH compounds in the standard calibration mixture injection, the GC/FID was checked daily. PAH mixture standards contained 15 compounds (Supelco, Inc., St. Louis, MO, USA) were used for preparation of the calibration curve to quantify the concentrations of the target PAH compounds. For all measured PAHs, the relative standard deviation of the replicated analyses of the calibration standard ranged from 4% to 6%. The average recovery of each PAH ranged from 70% to 108% for the 15 measured PAH compounds. The detection limit of each PAH compounds ranged from 0.048 ng/g to 0.376 ng/g.

The target PAH compounds in the surface street dust included naphthalene (NA), acenaphthylene (ACY), acenaphthene (ACE), fluorene (FLU), phenanthrene (PHE), anthracene (ANT), fluoranthene (FLT), pyrene (PYR), benzo(a)anthracene (BaA), chrysene (CRY), benzo(b)fluoranthene (BbF), benzo(a)pyrene (BaP), dibenzo(a,h)-anthracene (DBA), benzo(ghi)perylene (BGP), and indeno (1, 2, 3, -cd)pyrene (IND). According to the number of aromatic rings, PAH compounds in the surface street dust were classified into two aromatic rings (NA), three aromatic rings (ACY, ACE, FLU, PHE and ANT), four aromatic rings (FLT, PYR, BaA and CRY), five aromatic rings (BbF, BaP and DBA) and six aromatic rings (IND and BGB). The two and three rings PAH compounds were classified as low molecular weight (LMW) PAH compounds and four to six rings PAH compounds were classified as high molecular weight (HMW) PAH compounds.

### 2.5. Identification of the Possible Sources of PAH Compounds

The composition patterns of the measured PAHs varies significantly when they are derived from different sources. So, the concentration ratios of some individual PAH compounds can be used to give useful information about their source. PAHs are emitted from both petrogenic and pyrolytic sources. Concentration ratios of some individual PAH compounds like PHE/ANT, FLT/PYR, BaA/CRY, BaP/BGP, IND/(IND + BGP), FLU/ (FLU + PYR), ANT/(ANT + PHE), FLT/(FLT + PYR), LMW-PAHs/HMW-PAHs were used to determine the possible PAH emission sources [42,57,58,59,60,61,62,63].

### 2.6. Potential Health Risk Assessment of PAH Compounds

The toxic equivalent factor (TEF), carcinogenic PAHs (CPAHs) determination and the Incremental Lifetime Cancer Risk (ILCR) were used to estimate the potential risks of PAH compounds [8,14,44,64,65,66,67].

#### 2.6.1. Carcinogenic Potency of PAHs (BaP_equi_)

According to the TEFs proposed by Nisbet and LaGoy [64], the quantities of carcinogenic potency of PAH compounds in the surface street dust were calculated as benzo(a)pyrene equivalence (BaP_equi_). The reference chemical was BaP, the most toxic PAH and was assigned a value of one [68,69]. BaP_equi_ was calculated by using the following equation:

BaP_equi_ (ng/g) = C × TEF (1)

where, C is the concentration of individual PAH compounds; TEF is the corresponding individual equivalency factor for PAH compounds [64]. Then, the cancer potency of the total PAHs in the surface street dust was estimated by the summation of calculated cancer potency relative to BaP for all PAHs.

#### 2.6.2. Carcinogenic PAHs (CPAHs) Determination

According to the International Agency for Research on Cancer [70] and USEPA [71], carcinogenic PAH compounds (CPAHs) of the 15 PAHs examined in this study are BaA + CRY + BbF + BaP + DBA + IND. The CPAHs was calculated as the percentage composition of carcinogenic PAHs to other PAHs [14].

#### 2.6.3. Incremental Lifetime Cancer Risk (ILCR)

To evaluate the health risk assessment for children and adults exposed to PAHs in surface street dust, the USEPA standard models (residential scenario) [8,44,65,66,67] were used. Local residents are exposed to PAHs in the surface street dust through three main exposure pathways including: (1) direct ingestion, (2) inhalation through mouth and nose, and (3) dermal absorption. The carcinogenic risk was calculated for each PAH compounds in street dust by the summation of the individual risks calculated for the three exposure pathways. The cancer risk for total PAHs carcinogenic risk was computed by summing the individual PAH risks for each PAH compounds for the three exposure pathways. BaP_equi_ was used for risk assessment calculation. ILCR in terms of inhalation, dermal contact and direct ingestion was calculated based on the following equations:

(2) $${\mathrm{ILCR}}_{\mathrm{ingestion}}=\frac{\mathrm{Cs}\;x\;\left\{{\mathrm{CSF}}_{\mathrm{ingestion}}\times \sqrt[{3}]{\left(\frac{\mathrm{BW}}{70}\right)}\right\}\times {\mathrm{IR}}_{\mathrm{ing}.}\times \mathrm{EF}\times \mathrm{ED}}{\mathrm{BW}\times \mathrm{AT}\times 10^{6}}$$

(3) $${\mathrm{ILCR}}_{\mathrm{inhalation}}=\frac{\mathrm{Cs}\times \left\{{\mathrm{CSF}}_{\mathrm{inhalation}}\times \sqrt[{3}]{\left(\frac{\mathrm{BW}}{70}\right)}\right\}\times {\mathrm{IR}}_{\mathrm{inhal}.}\times \mathrm{EF}\times \mathrm{ED}}{\mathrm{BW}\times \mathrm{AT}\times \mathrm{PEF}}$$

(4) $${\mathrm{ILCR}}_{\mathrm{dermal}}=\frac{\mathrm{Cs}\times \left\{{\mathrm{CSF}}_{\mathrm{dermal}}\times \sqrt[{3}]{\left(\frac{\mathrm{BW}}{70}\right)}\right\}\times \mathrm{SA}\times \mathrm{AF}\times \mathrm{ABS}\times \mathrm{EF}\times \mathrm{ED}}{\mathrm{BW}\times \mathrm{AT}\times 10^{6}}$$

(5) $$\mathrm{Carcinogenic}\;\mathrm{risk}={\mathrm{ILCR}}_{\mathrm{ingestion}}+{\mathrm{ILCR}}_{\mathrm{inhalation}}+{\mathrm{ILCR}}_{\mathrm{dermal}}$$

where the ILCR is incremental lifetime cancer risk, CS the concentration of the PAH compounds in surface street dust based on toxic equivalent of BaP using the TEF, CSF the carcinogenic slope factor (mg/kg/day), BW the body weight (kg), AT the average life span (years), EF the exposure frequency (day/year), ED the exposure duration (years), IR_Inhalation_ the inhalation rate (m^3^/day), IR_Ingestion_ the soil intake rate (mg/day), SA the dermal surface exposure (cm^2^), AF the dermal adherence factor (mg/cm^2^/h), ABS the dermal adsorption fraction and PEF the particle emission factor (m^3^/kg). PEF is the particle emission factor (m^3^/kg) [8,44,67]. The detailed description about the values of exposure factors for children and adults applied to the above models (Equations (2)–(4)) in the present study are given in Table 1.

## 3. Results and Discussion

### 3.1. Concentration of PAH Compounds in Surface Street Dusts

The average concentrations of the individual and total PAH compounds in the surface street dusts collected from the different land use pattern of Jeddah and a rural area of Hada Al Sham are shown in Figure S1. For the street dust of Jeddah, the average concentrations of the individual PAH compounds in descending order were BbF > BGP > CRY > DBA > BaP > BaA > FLT > IND > PYR > PHE > ANT > ACE > FLU > NA > ACY (Figure S1a). The concentration values ranged from 81.44 ng/g (ACY) to 410.00 ng/g (BbF). Figure S1b shows the average concentrations of different categories of PAH compounds based on aromatic ring number. PAHs with five aromatic rings represented the highest levels in surface street dust of Jeddah. The average concentrations were 1051.30 ng/g for five aromatic rings, 1040.20 ng/g for four aromatic rings, 643.74 ng/g for six aromatic rings, 502.32 ng/g for three aromatic rings and 83.28 ng/g for two aromatic rings. Meanwhile, PAH compounds with five aromatic rings showed the highest concentration (95.10 ng/g) followed by six aromatic rings (55.00 ng/g), four aromatic rings (53.60 ng/g), three aromatic rings (17.05 ng/g) and two aromatic rings (2.20 ng/g) in surface street dust of RUA. The individual and total PAH compounds concentrations in the surface street dusts of Jeddah exceeded the RUA values. Their mean values were 37.85, 28.10, 28.02, 39.82, 26.67, 28.70, 44.76, 36.64, 26.69, 10.79, 11.42, 10.51, 11.14, 10.47, 12.56, 14.89 fold higher than those in the RUA for NA, ACY, ACE, FLU, PHE, ANT, FLT, PYR, BaA, CRY, BbF, BaP, DBA, IND, BGP and total PAHs, respectively. This indicates that the PAH compounds in the street dust might be derived mainly from anthropogenic sources and they tend to accumulate in surface street dust particles that can be used as indicator of environmental pollution in urban areas [8,11,13,44].

In the present study, the average concentration of total PAHs in surface street dust of Jeddah (3320 ng/g) was higher than that found in the Bushehr, Iran (1116 ng/g [67]), Kumas, Ghana (2571 ng/g [75]), Mahshahr, Iran (769.6 ng/g [76]), Hanoi, Vietnam (1900 ng/g [77]), Bangalore, India (1100 ng/g [77]), New Delhi, India (1100 ng/g [77]), Bangkok, Thailand (1100 ng/g [78]), Niteroi, Brazil (434–1247 ng/g [79]) and Lahore, Pakistan (120–1000 ng/g [80]). On the other hand, PAH pollution level in surface street dust of Jeddah was lower than those of Xuzhou, China (6616 ng/g [81]), Beijing, China (3700 ng/g [82]), Xi’an, China (15767 ng/g [26]), Guangzhou, China (4800 ng/g [11]), Dalian, China (7460 ng/g [2]), Birmingham, UK (12,560–93,700 ng/g [80]) and Pasadena, USA (58,680 ng/g [1]). These results indicate that PAHs concentrations in street dust samples vary around the world. These variations might be referred to the differences in traffic level and intensity of human activities, technologies employed, frequency of city street cleaning and local meteorological conditions such as rains which can remove the contaminants from street dust [6,11,14,41].

The wide variations of PAHs concentrations in street dust of urban areas arise from the different characteristics of the sampling areas such as location, traffic density, and type of vehicle. The spatial variations of the individual and total PAHs concentrations in surface street dusts from different land use patterns of Jeddah are shown in Table 2. The individual and total PAHs concentration levels showed variation with different land use pattern. The highest concentrations of the individual PAH compounds in surface street dust were found in TRA, and the lowest levels in REA (Table 2). Moreover, the highest concentrations of total PAHs were found in TRA (4980 ng/g) followed by URA (4150 ng/g), MCRA (3320 ng/g), SUA (2490 ng/g) and REA (1660 ng/g) (Table 2). The observed high values of the PAH compounds in the surface street dust in TRA indicate that the TRA areas may be a reservoir of PAH compounds arising from heavy traffics and human activities, since TRA in Jeddah cover major highway, parking areas, roundabout and crossroads with the highest traffic volumes. Anthropogenic activities contribute to PAHs accumulation in road dust [82]. Concerning road dusts from city centres, vehicle exhausts is the major source of pyrogenic PAHs [3,79,83]. Traffic exhausts, tire wear, resuspended soils dust, asphalt, and power plants are the main sources of PAH compounds in urban areas [84,85,86]. The minimum level of PAH compounds in the present study was found in REA. This might be due to its land use type and low anthropogenic activities [8]. Generally, the spatial variations of the concentrations of PAH compounds in different land use types might be attributed to the distinctive artificial activities in each land use type that emit PAH compounds deposited in the surface street dust.

### 3.2. Profile of PAH Compounds in Surface Street Dust

The relative contribution of the individual PAH compounds to the total PAHs concentrations in surface street dust of MCRA, REA, TRA, URA and SUA, were nearly similar. Generally, they increased with increasing molecular weight of PAH compounds. BbF, BGP, CRY, DBA and BaP showed relatively higher contribution, while NA, ACY, ACE and FLU were the least contributing. This similarity in contribution of the individual PAH compounds indicates that the study areas do share a common source of vehicle emission [11,87].

**Figure 2:**
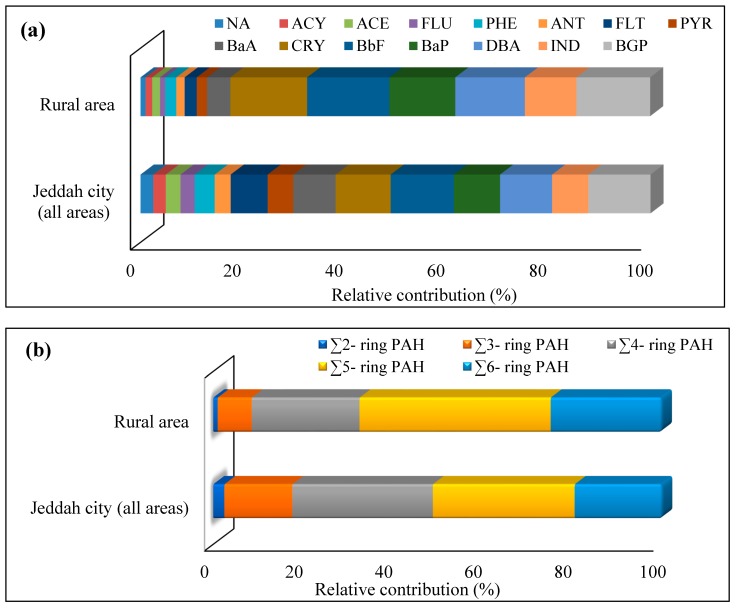
Relative contribution of each individual PAH compound and different categories of PAHs based on aromatic ring number to the total PAHs concentrations in street dusts collected from all areas of Jeddah city and a rural area (RUA) of Hada Al Sham: (**a**) individual PAH compounds and (**b**) two to six-ring PAHs.

Figure 2 shows the relative contribution of each individual PAH compound and different categories of PAHs based on aromatic ring number to the total PAHs concentrations in street dusts collected from all areas of Jeddah city and rural area. PAH compounds with four and six aromatic rings were the dominant in the surface street dust of Jeddah. The contribution of high molecular weight (HMW)-PAHs (four to six aromatic rings) (82.40%) was higher than that of low molecular weight (LMW) ones (two to three aromatic rings) (17.60%). This indicates the tendency of HMW-PAHs to adhere to street dust [11]. Moreover, the high contribution of HMW-PAHs suggests that they mostly come from pyrogenic sources [88] such as petroleum fuels combustion [4] and vehicular emissions [75]. The PAHs speciation in gasoline vehicle soot is enriched by HMW PAHs [78].

In surface street dust of RUA, the relative contribution of the individual PAH compounds to the total PAHs concentrations indicated that BbF, CRY, BGP, DBA, INSD and BaP were the predominant (Figure 2a). Based on aromatic ring number of PAH compounds, PAHs with five aromatic rings were the dominant (42.60%), followed by six aromatic rings (24.70%), four aromatic rings (24.03%), three aromatic rings (7.60%) and two aromatic rings (0.99%) (Figure 2b). High contribution of HMW-PAHs to the total PAHs concentrations in RUA suggests that PAHs mostly come from pyrogenic sources, which means that the rural background area received PAHs from the surrounding areas and predominantly was related to the traffic emissions. RUA at Hada Al Sham is far from traffic density. Therefore, the measured concentrations of PAHs in this rural area might result from the diffusion and dispersion of PAHs produced from the traffic density in the surrounding areas. Lee et al. [89] reported that high carbon content and more PAHs adsorbed in the young aerosol are emitted by the automobile exhaust. They added that the PAHs shift from the particle phase to gas phase in order to reach thermodynamic equilibrium during the transportation process from traffic to rural atmosphere. In the present study, the contribution of LMW-PAHs to the total PAHs concentrations in surface street dust of Jeddah (17.64%) was higher than that found in surface street dust of RUA (8.59%). This indicates that the time required for the transportation of the PAHs allows significant decrease the LMW-PAHs concentrations in particulate phase, and consequently their contribution to the total PAHs concentrations in street dust of the RUA decreased.

### 3.3. Possible Sources of PAH Compounds in Surface Street Dust

**Figure 3:**
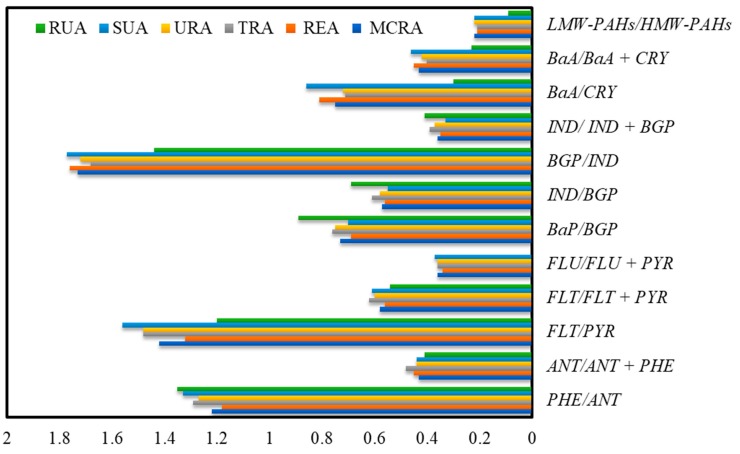
Diagnostic concentration ratios of selected PAH compounds in surface street dust collected from different land use patterns of Jeddah and a rural area (RUA) of Hada Al Sham.

To distinguish between both sources, LMW-PAHs/HMW-PAHs, ANT/(ANT + PHE), BaA/(BaA + CRY), FLU/(FLU+ PYR), IND/(IND + BGP), PHE/ANT and FLT/PYR concentration ratios are used. A LMW-PAHs/HMW-PAHs ratio > 1 indicates petrogenic source, while the ratio < 1 refers to pyrogenic sources [24]. An ANT/(ANT + PHE) ratio < 0.1 reflects a petroleum sources, while a ratio > 0.1 suggests a combustion source [62]. The ratios of PHE/ANT ˂ 10 and FLT/PYR ˃ 1.00 indicate that PAH compounds are emitted from pyrogenic source, while the ratios PHE/ANT ˃ 15.00 and FLT/PYR ˂ 1.00 suggest petrogenic origins of PAH compounds [90]. Similarly, Flu/(Flu + PYR) ratio < 0.4 indicates a petroleum source, ratios between 0.4 and 0.5 reveal a liquid fossil fuel combustion source, and a ratio > 0.5 characterizes biomass and coal combustion [62]. To identify the traffic sources, IND/BGP, BaA/CRY and BaP/BGP concentration ratios are used. The IND/BGP ratio for gasoline engines is about 0.40 and approaches 1.00 for diesel engine [91]. BaA/CRY concentration ratios range from 0.28 to 1.20 for gasoline engines and from 0.17 to 0.36 for diesel engines [25,92]. BaP/BGP concentration ratios higher than 0.60 also refer to the presence of traffic emissions [93]. IND/(IND + BGP) and BaA/(BaA + CRY) ratios are used to characterize the nature of potential PAH emission sources [62]. Ratios of IND/(IND + BGP) < 0.2 and BaA/(BaA + CRY < 0.2 indicate petroleum and petrogenic sources, respectively. BaA/(BaA + CRY) ratios between 0.2–0.35 and IND/(IND + BGP) ratios between 0.2–0.5 suggest that PAHs originate from petroleum combustion. To assess the presence of PAHs from fossil fuels inputs, ANT/ (ANT + PHE) concentration ratio is used [62]: ANT/ (ANT + PHE) concentration ratios ˂ 0.1 suggest non-burned fossil fuel inputs, while the combustion sources may prevail if the ratios are ˃ 0.1. Figure 3 shows the calculated diagnostic concentration ratios of the selected PAH compounds in surface street dust collected from different land use patterns of Jeddah and RUA of Hada Al Sham. PHE/ANT, FLT/PYR, ANT/(ANT + PHE), LMW-PAHs/HMW-PAHs, IND/(IND+BGP), BaA/(BaA + CRY), FLU/(FLU+PYR), BaP/BGP, IND/BGP, BaA/CRY concentration ratios indicated the main sources of PAH compounds in street dust samples of the study areas is from pyrogenic, mainly traffic emission.

PAHs concentrations in surface street dust can be influenced not only by local sources but also by long-distance transport sources. The ratio of a more reactive PAH to less reactive PAH can be used to give information about the photochemical degradation of PAH compounds and the aging of air mass [94,95]. BaA photodegradation is very fast and easier than its isomer CRY during their transportation [14,96], To estimate the distance of potential PAHs sources, BaA/CRY ratio is used as a diagnostic indicator to assess the origin of PAH compounds. A lower BaA/CRY ratio due to the photodegradation and oxidized degradation of most BaA means that the PAHs sources may be far away from the urban areas, whereas a higher ratio suggests that those PAHs might emit mostly from local sources [97].

Lohmann et al. [94] reported that a BaA/CRY ratio higher than 0.40 suggests that the PAHs pollution is freshly emitted and photochemical processing of the air mass is relatively lower, whereas a BaA/CRY ratio lower than 0.40 indicates that the major sources of PAHs are not local or the air masses are aged. In the present study, the mean concentration ratios of BaA/CRY were 0.75 in MCRA, 0.81 in REA, 0.71 in TRA, 0.72 in URA and 0.86 in SUA (Figure 3), which suggest that PAHs in street dusts of Jeddah come mainly from emission of local sources and the air masses are not aged. Liu et al. [4] reported that the BaA/CRY ratios of Shanghai road dust ranged from 0.6–1.4, which indicate that Shanghai might be close to its potential sources of dusts. On the other hand, the BaA/CRY ratio in the present study was 0.30 in RUA (Figure 3). This indicates that the sources of PAHs in RUA might not be local and transported from the surrounding urban areas. This result is in agreement with Mai et al. [97] who found that the BaA/CRY ratios in street dust ranged from 0.2–0.3 and suggested that Macao street dust might be transported from distant areas.

FLT, PYR, BaA, CRY, BbF, BaP, IND and BGP are eight combustion related non-alkylated PAH compounds (CPAHs). The ratio of the concentration of CPAHs to the total concentration of PAH compounds (CPAHs/total PAHs) is used to distinguish the possible emissions of PAHs from mobile versus stationary combustion sources. For mobile sources, the value of CPAHs/total PAHs ratio was found to be less than one [1]. In the present study, the average CPAHs/total PAHs concentration ratios at Jeddah (0.72) indicates that the mobile sources are the principal PAHs contributor to surface street dusts of Jeddah city.

### 3.4. Risk Assessment of PAHs in Surface Street Dust

#### 3.4.1. Carcinogenic PAHs (CPAHs) Determination

The total concentration of CAPAHs in the surface street dusts of different land use pattern of Jeddah were 1918.7 ng/g (MCRA), 957.35 ng/g (REA), 2887.05 ng/g (TRA), 2405.88 ng/g (URA) and 1433.53 ng/g (SUA). These concentrations exceeded the RUA values. Their mean values were 11.90 (MCRA), 5.94 (REA), 17.90 (TRA), 14.92 (URA) and 8.89 (SUA) fold higher than those in the RUA. The relative contribution of the individual CAPAHs to the total PAHs concentrations in surface street dust of MCRA, REA, TRA, URA and SUA, were nearly similar. Generally, BbF was the dominant, followed CRY, DBA, BaP, BaA and IND. Moreover, the average total concentration of CAPAHs accounted for 57.84% of the total PAHs in street dusts from different areas of Jeddah.

#### 3.4.2. Carcinogenic Potency of PAHs Based on BaP_equi_

BaP is known to be the most potent carcinogenic PAH compound [98] and is used as a good index for whole PAH carcinogenicity [99]. It is also used as a marker for total PAHs exposure in the environment [100]. The levels of carcinogenic potency of each PAH compounds in the surface street dust were calculated and assessed on the basis of its BaP_equi_ concentration using TEFs (Table 3). BaP_equi_ concentrations of the individual PAH compounds in the surface street dust ranged from 0.07 ng BaP_equi_/g (NA) to 338.68 ng BaP_equi_/g for DBA in MCRA, 0.04 ng BaP_equi_/g (NA and ACY) to 169.29 ng BaP_equi_/g (DBA) in REA, 0.12 ng BaP_equi_/g (ACY) to 507.87 ng BaP_equi_/g (DBA) in TRA, 0.10 ng BaP_equi_/g (ACY) to 423.23 ng BaP_equi_/g (DBA) in URA, 0.06 ng BaP_equi_/g (NA and ACY) to 253.94 ng BaP_equi_/g (DBA) in SUA and 0.002 ng BaP_equi_/g (NA and FLU) to 30.40 ng BaP_equi_/g (DBA) in RUA (Table 3). In addition, the total carcinogenic potency (total PAHBaP_equi_) for the total PAHs were 741, 369, 1119, 933, 552 and 67 ng BaP_equi_/g in street dust of MCRA, REA, TRA, URA, SUA and RUA, respectively. Carcinogenic 6 PAH compounds (BaA, CRY, BbF, BaP, DBA and IND) were the major contributors to total PAHBaP_equi_ in street dust samples. Their contribution to the total PAHBaP_equi_ decreased in the order of DBA > BaP > BbF > BaA >IND > CRY for MCRA, REA, TRA, URA, SUA, and DBA > BaP > BbF > IND >BaA > CRY for RUA. The average value of the total PAHBaP_equi_ in street dust of Jeddah (742.7 ng BaP_equi_ /g) was lower than that found in street side soil of Sanghai, China (892 ng BaP_equi_/g [101]), traffic soil of Delhi, India (1009 ng BaP_equi_/g [58]). Whereas total PAHBaP_equi_ values in street dusts of Jeddah was higher than those found in street dust of Guwahati city (357.7 ng BaP_equi_/g [14]), Lanzhou, China (300 ng BaP_equi_/g [44]), street dust of Asansol city, India (661 ng BaP_equi_/g [8]).

#### 3.4.3. Incremental Lifetime Cancer Risk (ILCR)

The estimated ILCR value (based on the values of TEF and CSF) was used to identify the potential cancer risks for human exposure to PAH pollution sources. Chen and Liao [31] and Jiang et al. [44] reported that the values of ILCR between 10^−6^ and 10^−4^ indicate potential risk, ILCR ≤ 10^−6^ indicate virtual safety, and ILCR > 10^−4^ indicate a potentially high risk. Based on the calculated ILCR values for the ingestion (ILC_ingesion_), inhalation (ILCR_inhalation_), dermal (ILCR_dermal_) pathways and total cancer risk (∑ILCR_ingestion_ + ILCR_inhalation_ + ILCR_dermal_) for children and adults exposed to PAHs in street dust (Tables S1 and S2), the sequence of cancer risk of the different areas was TRA > URA > MCRA > SUA > REA > RUA. This variation in cancer risks among the six land use areas indicates the great effects of source emissions of PAHs on the health risk. Concerning the PAHs, BaA, CRY, BbF, BaP, DBA, IND and BGP showed high ILCR_ingestion_, ILCR_inhalation_ and ILCR_dermal_ for children and adults compared with other PAH compounds in the different study areas. The average cancer risk values of the individual PAH compounds in street dust of Jeddah for children and adults were in the order of DBA > BaP > BbF > BaA > IND > BGP > CRY > ANT > FLT > PYR > PHE > ACE > FLU > ACY > NA. Results reveal that potential risk for both children and adults was found only for exposure to BaP and DBA in street dust in different land areas in Jeddah, since cancer risk (∑ILCRs) values were between 10^−6^ and 10^−4^ [31,44]. Similarly, the average ILCR_ingestion_, ILCR_dermal_ and cancer risk values based on average total PAHBaP_equi_ concentrations in Jeddah were found between 10^−6^ and 10^−4^ (Table 4), indicating a potential risk. Table 4 also shows that the ILCR_dermal_ and ILCR_ingestion_ values for children and adults are higher than that of ILCR_inhalation_, indicating that the inhalation pathway for PAHs exposure is of minor importance. This is confirmed by Jiang et al. [44], Soltani et al. [41], Keshavarzi et al. [67], Gope et al. [8] and Škrbić, et al. [43]. The cancer risk values of direct ingestion for children were higher than the corresponding risk values of ingestion for adults at the different study areas, indicating that the young children were the most sensitive subpopulation due to their hand-to-mouth activity [8,11,44].

## 4. Conclusions

The present study reports the status, source and health risk assessment of PAHs in the surface street dust from different land use patterns in Jeddah and a rural area at Hada Al Sham. The concentration of ∑PAHs in street dust indicates that PAH compounds might be derived mainly from anthropogenic sources and tend to accumulate in surface street dust particles. High PAHs levels were observed in traffic, urban and mixed commercial/residential areas and low levels in residential area. This was referred to the distinctive artificial activities in each land. High molecular weight—PAHs were dominant in the surface street dust. The main source of PAH compounds in the study areas is pyrogenic, mainly traffic emission. Moreover, PAHs in street dusts of Jeddah come mainly from emission of local sources and the air masses are not aged, whereas the sources of PAHs in Hada Al Sham might not be local but transported from the surrounding urban areas. Based on the calculated ILCR_ing._, ILCR_inh._, ILCR_dermal_ and cancer risks for children and adults exposed to PAHs in street dust, the sequence of cancer risk of the different areas was TRA > URA > MCRA > SUA > REA > RUA. Moreover, the ILCR_dermal_ and ILCR_ingestion_ values for children and adults are higher than that of ILCR_inhalation_, indicating that the inhalation pathway for PAHs exposure is of minor importance. Potential risk for both children and adults was found only for exposure to BaP and DBA in street dust in different land areas in Jeddah. The average ILCR_ingestion_, ILCR_dermal_ and cancer risk values based on average total PAHBaP_equi_ concentrations for children and adults exposed to PAHs in street dust of different areas in Jeddah were found between 10^−6^ and 10^−4^, indicating a potential risk.

## Figures and Tables

**Table 1:** Values of exposure parameters and factors used for the incremental lifetime cancer risk assessment.

Exposure Factors	Description	Unit	Adult	Child	Reference
IR_ingestion_	Ingestion rate	mg/day	100	200	USEPA [72]
SA	Exposed skin area	cm^2^/day	5700	2800	USEPA [72]
Af_soil_	Skin adherence factor	mg/cm^2^	0.07	0.2	USEPA [72]
EF	Exposure frequency	days/year	365	365	Kumar et al. [73]
ED	Exposure duration	year	24	6	USEPA [72]
BW	Body weight	kg	70	15	USEPA [65]
AT	Averaging time (70 years × 365 days/year)	days	25,550	25,550	Ferreira-Baptista and De-Miguel [74]
ABS	Dermal adsorption fraction	unitless	0.13	0.13	USEPA [72]
IR_inhalation_	Inhalation rate	m^3^/day	20	10	Soltani et al. [41]
PEF	Particle emission factor	m^3^/kg	1.36 × 10^9^	1.36 × 10^9^	USEPA [72]
CSF_ingestion_	Carcinogenic slope factor for ingestion	mg/kg/day	7.3	7.3	Peng et al. [20]
CSF_inhalation_	Carcinogenic slope factor for inhalation	mg/kg/day	3.85	3.85	Peng et al. [20]
CSF_dermal_	Carcinogenic slope factor for dermal	mg/kg/day	25	25	Peng et al. [20]

**Table 2:** The concentrations (ng/g) of the individual PAH compounds and different categories of PAHs based on aromatic ring number in street dusts collected from the different areas of Jeddah city.

PAH Compounds	MCRA		REA		TRA		URA		SUA	
	Mean	SD	Mean	SD	Mean	SD	Mean	SD	Mean	SD
NA	83.88	18.44	43.44	8.87	127.32	18.83	105.60	17.23	56.16	12.35
∑2- ring PAH ^a^	83.88	18.44	43.44	8.87	127.32	18.83	105.60	17.23	56.16	12.35
ACY	81.24	20.67	40.12	9.38	121.36	19.37	101.30	16.16	63.18	13.89
ACE	95.28	24.82	46.14	9.39	142.42	25.44	118.85	20.23	73.71	16.21
FLU	93.60	23.28	45.80	10.33	135.4	22.23	113.00	22.25	70.20	15.44
PHE	129.04	32.27	63.52	11.49	196.56	33.34	163.80	30.33	100.48	21.61
ANT	104.96	25.30	53.48	9.99	154.44	29.59	128.70	20.21	75.02	16.98
∑3- ring PAH ^b^	504.12	120.72	249.06	50.11	750.18	123.38	625.65	108.59	382.59	84.14
FLT	238.88	56.83	115.44	22.77	364.32	58.77	303.60	50.34	186.16	40.06
PYR	167.68	38.70	87.84	16.38	245.52	38.67	204.60	37.67	118.76	27.00
BaA	273.00	62.66	142.00	24.88	396.00	67.55	330.00	56.45	220.00	43.54
CRY	360.60	86.73	174.80	35.19	554.40	87.34	462.00	77.65	255.20	60.96
∑4- ring PAH ^c^	1040.16	246.15	520.08	102.18	1560.24	251.58	1300.20	221.42	780.12	171.56
BbF	410.00	95.33	205.00	38.68	615.00	95.44	512.50	89.24	307.50	67.62
BaP	300.90	70.35	148.45	28.89	460.35	70.45	383.63	67.33	220.18	50.62
DBA	338.58	79.39	169.29	32.13	507.87	88.56	423.23	70.40	253.94	55.84
∑5- ring PAH ^d^	1049.48	249.77	522.74	103.68	1583.22	255.29	1319.35	224.68	781.61	174.08
IND	235.62	53.44	117.81	22.45	353.43	55.66	294.53	51.55	176.72	38.86
BGP	409.92	93.88	206.96	37.49	605.88	99.29	504.90	85.67	312.94	66.62
∑6- ring PAH ^e^	645.54	151.34	324.77	62.82	959.31	154.68	799.43	136.14	489.66	105.48
∑PAHs	3320.18	765.92	1660.09	306.77	4980.27	498.88	4150.23	706.76	2490.14	547.60

^a^ Total two-aromatic rings PAH compounds, ^b^ Total three-aromatic rings PAH compounds, ^c^ Total four-aromatic rings PAH compounds, ^d^ Total five-aromatic rings PAH compounds, ^e^ Total six-aromatic rings PAH compounds.

**Table 3:** BaP equivalent concentration (ng BaP_equi_/g) for the individual PAH compounds in street dusts collected from the different areas of Jeddah city and a rural area (RUA) of Hada Al Sham.

PAH Compounds	TEF ^a^	MCRA	REA	TRA	URA	SUA	Jeddah City (All Areas)	RUA
		Mean ± SD	Mean ± SD	Mean ± SD	Mean ± SD	Mean ± SD	Mean ± SD	Mean ± SD
NA	0.001	0.07 ± 0.02	0.04 ± 0.01	0.13 ± 0.02	0.11 ± 0.02	0.06 ± 0.01	0.08 ± 0.02	0.002 ± 0.001
ACY	0.001	0.08 ± 0.02	0.04 ± 0.01	0.12 ± 0.02	0.10 ± 0.02	0.06 ± 0.01	0.08 ± 0.02	0.003 ± 0.001
ACE	0.001	0.10 ± 0.02	0.05 ± 0.01	0.14 ± 0.03	0.12 ± 0.02	0.07 ± 0.02	0.10 ± 0.02	0.003 ± 0.001
FLU	0.001	0.09 ± 0.02	0.05 ± 0.01	0.14 ± 0.02	0.11 ± 0.02	0.07 ± 0.02	0.09 ± 0.02	0.002 ± 0.001
PHE	0.001	0.13 ± 0.03	0.06 ± 0.01	0.20 ± 0.03	0.16 ± 0.03	0.10 ± 0.02	0.13 ± 0.03	0.005 ± 0.001
ANT	0.010	1.05 ± 0.25	0.53 ± 0.10	1.54 ± 0.30	1.29 ± 0.02	0.75 ± 0.17	1.03 ± 0.21	0.036 ± 0.008
FLT	0.001	0.24 ± 0.06	0.12 ± 0.02	0.36 ± 0.06	0.30 ± 0.05	0.19 ± 0.04	0.24 ± 0.05	0.005 ± 0.001
PYR	0.001	0.17 ± 0.04	0.09 ± 0.02	0.25 ± 0.04	0.20 ± 0.04	0.12 ± 0.03	0.16 ± 0.03	0.005 ± 0.001
BaA	0.100	27.3 ± 6.27	14.20 ± 2.49	39.60 ± 6.76	33.00 ± 5.65	22.00 ± 4.35	27.22 ± 5.41	1.020 ± 0.113
CRY	0.010	3.61 ± 0.87	1.75 ± 0.35	5.54 ± 0.87	4.62 ± 0.78	2.55 ± 0.61	3.61 ± 0.73	0.335 ± 0.037
BbF	0.100	41 ± 9.53	20.50 ± 3.87	61.50 ± 9.54	51.25 ± 8.92	30.75 ± 6.76	41.00 ± 7.96	3.590 ± 0.398
BaP	1.00	300.9 ± 70.35	148.45 ± 28.89	460.35 ± 70.45	383.63 ± 67.33	220.18 ± 50.65	302.70 ± 59.74	28.800 ± 3.197
DBA	1.00	338.58 ± 79.39	169.29 ± 32.13	507.87 ± 88.56	423.25 ± 70.40	253.94 ± 55.84	338.58 ± 66.69	30.400 ± 3.374
IND	0.100	23.56 ± 5.34	11.78 ± 2.25	35.34 ± 5.57	29.45 ± 5.16	17.67 ± 3.89	23.56 ± 4.57	2.250 ± 0.250
BGP	0.010	4.10 ± 0.94	2.07 ±0.37	6.06 ± 0.99	5.05 ±0.86	3.13 ± 0.67	4.08 ± 0.78	0.325 ± 0.036
Total carcinogenic potency		740.98 ± 170.93	369.02 ± 68.19	1119.14 ± 112.11	932.62 ±158.82	551.63 ± 121.31	742.68 ± 134.55	66.78 ± 7.419

^a^ TEF values from Nisbet and La Goy [64].

**Table 4:** Incremental lifetime cancer risks and cancer risks of the total PAH compounds for children and adults population living in different areas of Jeddah city and a rural area (RUA) of Hada Al Sham.

Area	Child				Adult			
	ILCR_ingestion_	ILCR_inhalation_	ILCR_dermal_	Cancer Risk	ILCR_ingestion_	ILCR_inhalation_	ILCR_dermal_	Cancer Risk
MCRA	3.7 × 10^−6^	7.2 × 10^−11^	4.61 × 10^−6^	8.3 × 10^−6^	2.65 × 10^−6^	2.05 × 10^−10^	4.71 × 10^−6^	7.35 × 10^−6^
REA	1.8 × 10^−6^	3.6 × 10^−11^	2.30 × 10^−6^	4.1 × 10^−6^	1.32 × 10^−6^	1.02 × 10^−10^	2.34 × 10^−6^	3.66 × 10^−6^
TRA	5.6 × 10^−6^	1.1 × 10^−10^	6.96 × 10^−6^	2.6 × 10^−5^	4.00 × 10^−6^	3.10 × 10^−10^	7.11 × 10^−6^	1.11 × 10^−5^
URA	4.7 × 10^−6^	9.0 × 10^−11^	5.80 × 10^−6^	1.0 × 10^−5^	3.33 × 10^−6^	2.58 × 10^−10^	5.92 × 10^−6^	9.25 × 10^−6^
SUA	2.8 × 10^−6^	5.4 × 10^−11^	3.43 × 10^−6^	6.2 × 10^−6^	1.97 × 10^−6^	1.53 × 10^−10^	3.50 × 10^−6^	5.47 × 10^−6^
Jeddah city (all areas)	3.7 × 10^−6^	7.2 × 10^−11^	4.62 × 10^−6^	8.3 × 10^−6^	2.65 × 10^−6^	2.06 × 10^−10^	4.72 × 10^−6^	7.37 × 10^−6^
RUA	3.3 × 10^−7^	6.5 × 10^−12^	4.15 × 10^−7^	7.5 × 10^−7^	2.38 × 10^−7^	1.85 × 10^−11^	4.24 × 10^−7^	6.62 × 10^−7^

## Supplementary Materials

The following are available online at http://www.mdpi.com/1660-4601/15/11/2397/s1. Figure S1: Comparison between the concentrations of each individual PAH compound and different categories of PAHs based on aromatic ring number in street dusts collected from all areas of Jeddah city and a rural area (RUA) of Hada Al sham: (a) individual PAH compounds and (b) two to six-ring PAHs, Table S1: Incremental lifetime cancer risks and cancer risks of the individual PAH compounds for children population living in different areas of Jeddah city and a rural area (RUA) of Hada Al Sham, Table S2: Incremental lifetime cancer risks and cancer risks of the individual PAH compounds for adults population living in the different areas of Jeddah city and a rural area (RUA) of Hada Al Sham.
