# Supplementary material for: Street Dust—Bound Polycyclic Aromatic Hydrocarbons in a Saudi Coastal City: Status, Profile, Sources, and Human Health Risk Assessment
Source: Int J Environ Res Public Health. 2018 Oct 29;15(11):2397. doi: 10.3390/ijerph15112397 (PMC6266921; doi:10.3390/ijerph15112397)
Supplement: Supplementary file 1 [file ijerph-15-02397-s001.pdf]

Supplementary Tables & Figures

Street Dust - Bound Polycyclic Aromatic Hydrocarbons in Saudi Coastal City: Status, Profile, Sources, and Human Health Risk Assessment

Ibrahim I. Shabbaj, Mansour A. Alghamdi* and Mamdouh I. Khoder

Department of Environmental Sciences, Faculty of Meteorology, Environment and Arid Land Agriculture, King Abdulaziz University, P.O. Box 80208, Jeddah 21589, Saudi Arabia; ishabbaj@kau.edu.sa (I.I.S.); mkhader@kau.edu.sa (M.I.K.)

***** Correspondence: mghamdi2@kau.edu.sa; Tel.: +966-550-50-0014

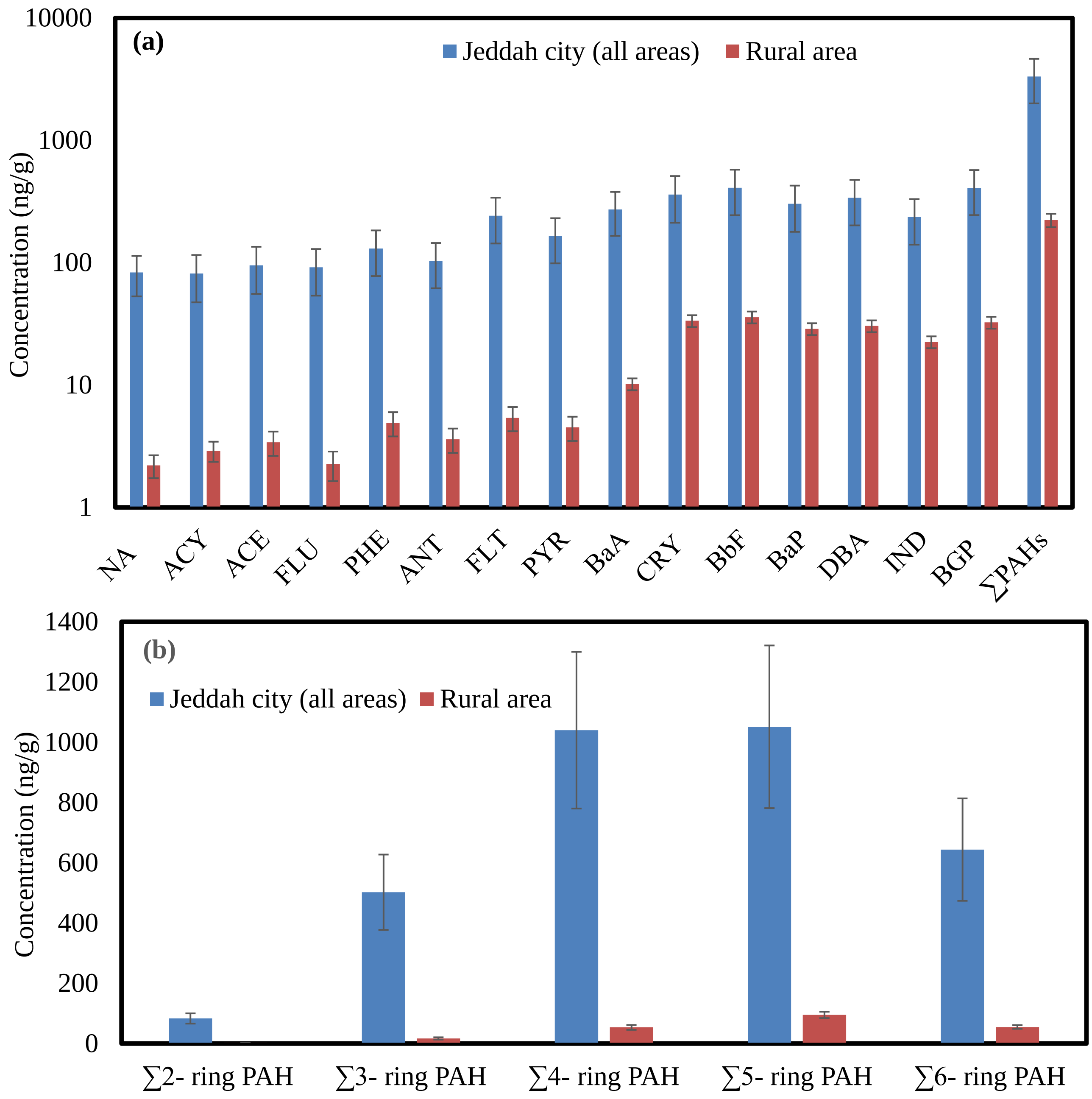

Figure S1. Comparison between the concentrations of each individual PAH compound and different categories of PAHs based on aromatic ring number in street dusts collected from all areas of Jeddah city and a rural area (RUA) of Hada Al sham: (a) individual PAH compounds and (b) two to six-ring PAHs.

Table S1. Incremental lifetime cancer risks and cancer risks of the individual PAH compounds for children population living in different areas of Jeddah city and a rural area (RUA) of Hada Al Sham

Risk	Area	PAH compounds														
		NA	ACY	ACE	FLU	PHE	ANT	FLT	PYR	BaA	CRY	BbF	BaP	DBA	IND	BGP
$ILCR_{ingestion}$	MCRA	4.2E-10	4.1E-10	4.8E-10	4.7E-10	6.4E-10	5.2E-09	1.2E-09	8.4E-10	1.4E-07	1.8E-08	2.0E-07	1.5E-06	1.7E-06	1.2E-07	2.0E-08
	REA	2.2E-10	2.0E-10	2.3E-10	2.3E-10	3.2E-10	2.7E-09	5.8E-10	4.4E-10	7.1E-08	8.7E-09	1.0E-07	7.4E-07	8.4E-07	5.9E-08	1.0E-08
	TRA	6.4E-10	6.1E-10	7.1E-10	6.8E-10	9.8E-10	7.7E-09	1.8E-09	1.2E-09	2.0E-07	2.8E-08	3.1E-07	2.3E-06	2.5E-06	1.8E-07	3.0E-08
	URA	5.3E-10	5.1E-10	5.9E-10	5.6E-10	8.2E-10	6.4E-09	1.5E-09	1.0E-09	1.6E-07	2.3E-08	2.6E-07	1.9E-06	2.1E-06	1.5E-07	2.5E-08
	SUA	2.8E-10	3.2E-10	3.7E-10	3.5E-10	5.0E-10	3.7E-09	9.3E-10	5.9E-10	1.1E-07	1.3E-08	1.5E-07	1.1E-06	1.3E-06	8.8E-08	1.6E-08
	RUA	1.1E-11	1.4E-11	1.7E-11	1.1E-11	2.4E-11	1.8E-10	2.7E-11	2.2E-11	5.1E-09	1.7E-09	1.8E-08	1.4E-07	1.5E-07	1.1E-08	1.6E-09
$ILCR_{inhalation}$	MCRA	8.1E-15	7.9E-15	9.2E-15	9.1E-15	1.3E-14	1.0E-13	2.3E-14	1.6E-14	2.6E-12	3.5E-13	4.0E-12	2.9E-11	3.3E-11	2.3E-12	4.0E-13
	REA	4.2E-15	3.9E-15	4.5E-15	4.4E-15	6.2E-15	5.2E-14	1.1E-14	8.5E-15	1.4E-12	1.7E-13	2.0E-12	1.4E-11	1.6E-11	1.1E-12	2.0E-13
	TRA	1.2E-14	1.2E-14	1.4E-14	1.3E-14	1.9E-14	1.5E-13	3.5E-14	2.4E-14	3.8E-12	5.4E-13	6.0E-12	4.5E-11	4.9E-11	3.4E-12	5.9E-13
	URA	1.0E-14	9.8E-15	1.2E-14	1.1E-14	1.6E-14	1.2E-13	2.9E-14	2.0E-14	3.2E-12	4.5E-13	5.0E-12	3.7E-11	4.1E-11	2.9E-12	4.9E-13
	SUA	5.4E-15	6.1E-15	7.1E-15	6.8E-15	9.7E-15	7.3E-14	1.8E-14	1.2E-14	2.1E-12	2.5E-13	3.0E-12	2.1E-11	2.5E-11	1.7E-12	3.0E-13
	RUA	2.1E-16	2.8E-16	3.3E-16	2.2E-16	4.8E-16	3.5E-15	5.2E-16	4.4E-16	9.9E-14	3.2E-14	3.5E-13	2.8E-12	2.9E-12	2.2E-13	3.2E-14
$ILCR_{dermal}$	MCRA	5.22E-10	5.05E-10	5.93E-10	5.82E-10	8.03E-10	6.53E-09	1.49E-09	1.04E-09	1.70E-07	2.24E-08	2.55E-07	1.87E-06	2.11E-06	1.47E-07	2.55E-08
	REA	2.70E-10	2.50E-10	2.87E-10	2.85E-10	3.95E-10	3.33E-09	7.18E-10	5.46E-10	8.83E-08	1.09E-08	1.28E-07	9.23E-07	1.05E-06	7.33E-08	1.29E-08
	TRA	7.92E-10	7.55E-10	8.86E-10	8.42E-10	1.22E-09	9.61E-09	2.27E-09	1.53E-09	2.46E-07	3.45E-08	3.83E-07	2.86E-06	3.16E-06	2.20E-07	3.77E-08
	URA	6.57E-10	6.30E-10	7.39E-10	7.03E-10	1.02E-09	8.01E-09	1.89E-09	1.27E-09	2.05E-07	2.87E-08	3.19E-07	2.39E-06	2.63E-06	1.83E-07	3.14E-08
	SUA	3.49E-10	3.93E-10	4.58E-10	4.37E-10	6.25E-10	4.67E-09	1.16E-09	7.39E-10	1.37E-07	1.59E-08	1.91E-07	1.37E-06	1.58E-06	1.10E-07	1.95E-08
	RUA	1.37E-11	1.80E-11	2.11E-11	1.40E-11	3.05E-11	2.24E-10	3.36E-11	2.80E-11	6.34E-09	2.08E-09	2.23E-08	1.79E-07	1.89E-07	1.40E-08	2.02E-09
Cancer risk	MCRA	9.4E-10	9.1E-10	1.1E-09	1.0E-09	1.4E-09	1.2E-08	2.7E-09	1.9E-09	3.1E-07	4.0E-08	4.6E-07	3.4E-06	3.8E-06	2.6E-07	4.6E-08
	REA	4.9E-10	4.5E-10	5.2E-10	5.1E-10	7.1E-10	6.0E-09	1.3E-09	9.8E-10	1.6E-07	2.0E-08	2.3E-07	1.7E-06	1.9E-06	1.3E-07	2.3E-08
	TRA	1.8E-09	1.9E-09	2.2E-09	2.1E-09	3.0E-09	2.3E-08	5.6E-09	3.7E-09	6.3E-07	8.4E-08	9.6E-07	7.1E-06	7.9E-06	5.5E-07	9.5E-08
	URA	1.2E-09	1.1E-09	1.3E-09	1.3E-09	1.8E-09	1.4E-08	3.4E-09	2.3E-09	3.7E-07	5.2E-08	5.7E-07	4.3E-06	4.7E-06	3.3E-07	5.7E-08
	SUA	6.3E-10	7.1E-10	8.3E-10	7.9E-10	1.1E-09	8.4E-09	2.1E-09	1.3E-09	2.5E-07	2.9E-08	3.4E-07	2.5E-06	2.8E-06	2.0E-07	3.5E-08
	RUA	2.5E-11	3.3E-11	3.8E-11	2.5E-11	5.5E-11	4.0E-10	6.1E-11	5.0E-11	1.1E-08	3.8E-09	4.0E-08	3.2E-07	3.4E-07	2.5E-08	3.6E-09

Table S2. Incremental lifetime cancer risks and cancer risks of the individual PAH compounds for adults population living in the different areas of Jeddah city and a rural area (RUA) of Hada Al Sham

Risk	Area	PAH compounds														
		NA	ACY	ACE	FLU	PHE	ANT	FLT	PYR	BaA	CRY	BbF	BaP	DBA	IND	BGP
$ILCR_{ingestion}$	MCRA	2.99E-10	2.90E-10	3.40E-10	3.34E-10	4.61E-10	3.75E-09	8.53E-10	5.99E-10	9.75E-08	1.29E-08	1.46E-07	1.07E-06	1.21E-06	8.41E-08	1.46E-08
	REA	1.55E-10	1.43E-10	1.65E-10	1.64E-10	2.27E-10	1.91E-09	4.12E-10	3.14E-10	5.07E-08	6.24E-09	7.32E-08	5.30E-07	6.04E-07	4.21E-08	7.39E-09
	TRA	4.55E-10	4.33E-10	5.08E-10	4.83E-10	7.02E-10	5.51E-09	1.30E-09	8.77E-10	1.41E-07	1.98E-08	2.20E-07	1.64E-06	1.81E-06	1.26E-07	2.16E-08
	URA	3.77E-10	3.62E-10	4.24E-10	4.03E-10	5.85E-10	4.59E-09	1.08E-09	7.30E-10	1.18E-07	1.65E-08	1.83E-07	1.37E-06	1.51E-06	1.05E-07	1.80E-08
	SUA	2.00E-10	2.26E-10	2.63E-10	2.51E-10	3.59E-10	2.68E-09	6.65E-10	4.24E-10	7.85E-08	9.11E-09	1.10E-07	7.86E-07	9.07E-07	6.31E-08	1.12E-08
	RUA	7.85E-12	1.04E-11	1.21E-11	8.03E-12	1.75E-11	1.29E-10	1.93E-11	1.61E-11	3.64E-09	1.20E-09	1.28E-08	1.03E-07	1.09E-07	8.03E-09	1.16E-09
$ILCR_{inhalation}$	MCRA	2.32E-14	2.25E-14	2.64E-14	2.59E-14	3.57E-14	2.91E-13	6.62E-14	4.64E-14	7.56E-12	9.99E-13	1.14E-11	8.33E-11	9.38E-11	6.53E-12	1.14E-12
	REA	1.20E-14	1.11E-14	1.28E-14	1.27E-14	1.76E-14	1.48E-13	3.20E-14	2.43E-14	3.93E-12	4.84E-13	5.68E-12	4.11E-11	4.69E-11	3.26E-12	5.73E-13
	TRA	3.53E-14	3.36E-14	3.95E-14	3.75E-14	5.44E-14	4.28E-13	1.01E-13	6.80E-14	1.10E-11	1.54E-12	1.70E-11	1.28E-10	1.41E-10	9.79E-12	1.68E-12
	URA	2.93E-14	2.81E-14	3.29E-14	3.13E-14	4.54E-14	3.56E-13	8.41E-14	5.67E-14	9.14E-12	1.28E-12	1.42E-11	1.06E-10	1.17E-10	8.16E-12	1.40E-12
	SUA	1.56E-14	1.75E-14	2.04E-14	1.94E-14	2.78E-14	2.08E-13	5.16E-14	3.29E-14	6.09E-12	7.07E-13	8.52E-12	6.10E-11	7.03E-11	4.90E-12	8.67E-13
	RUA	6.09E-16	8.03E-16	9.42E-16	6.23E-16	1.36E-15	9.97E-15	1.50E-15	1.25E-15	2.83E-13	9.28E-14	9.94E-13	7.98E-12	8.42E-12	6.23E-13	9.00E-14
$ILCR_{dermal}$	MCRA	5.33E-10	5.16E-10	6.05E-10	5.94E-10	8.19E-10	6.66E-09	1.52E-09	1.06E-09	1.73E-07	2.29E-08	2.60E-07	1.91E-06	2.15E-06	1.50E-07	2.60E-08
	REA	2.76E-10	2.55E-10	2.93E-10	2.91E-10	4.03E-10	3.40E-09	7.33E-10	5.58E-10	9.02E-08	1.11E-08	1.30E-07	9.43E-07	1.07E-06	7.48E-08	1.31E-08
	TRA	8.08E-10	7.71E-10	9.04E-10	8.60E-10	1.25E-09	9.81E-09	2.31E-09	1.56E-09	2.51E-07	3.52E-08	3.91E-07	2.92E-06	3.22E-06	2.24E-07	3.85E-08
	URA	6.71E-10	6.43E-10	7.55E-10	7.18E-10	1.04E-09	8.17E-09	1.93E-09	1.30E-09	2.10E-07	2.93E-08	3.25E-07	2.44E-06	2.69E-06	1.87E-07	3.21E-08
	SUA	3.57E-10	4.01E-10	4.68E-10	4.46E-10	6.38E-10	4.76E-09	1.18E-09	7.54E-10	1.40E-07	1.62E-08	1.95E-07	1.40E-06	1.61E-06	1.12E-07	1.99E-08
	RUA	1.40E-11	1.84E-11	2.16E-11	1.43E-11	3.11E-11	2.29E-10	3.43E-11	2.86E-11	6.48E-09	2.13E-09	2.28E-08	1.83E-07	1.93E-07	1.43E-08	2.06E-09
Cancer risk	MCRA	8.32E-10	8.06E-10	9.45E-10	9.29E-10	1.28E-09	1.04E-08	2.37E-09	1.66E-09	2.71E-07	3.58E-08	4.07E-07	2.99E-06	3.36E-06	2.34E-07	4.07E-08
	REA	4.31E-10	3.98E-10	4.58E-10	4.54E-10	6.30E-10	5.31E-09	1.15E-09	8.71E-10	1.41E-07	1.73E-08	2.03E-07	1.47E-06	1.68E-06	1.17E-07	2.05E-08
	TRA	1.26E-09	1.20E-09	1.41E-09	1.34E-09	1.95E-09	1.53E-08	3.61E-09	2.44E-09	3.93E-07	5.50E-08	6.10E-07	4.57E-06	5.04E-06	3.51E-07	6.01E-08
	URA	1.05E-09	1.00E-09	1.18E-09	1.12E-09	1.62E-09	1.28E-08	3.01E-09	2.03E-09	3.27E-07	4.58E-08	5.08E-07	3.81E-06	4.20E-06	2.92E-07	5.01E-08
	SUA	5.57E-10	6.27E-10	7.31E-10	6.96E-10	9.97E-10	7.44E-09	1.85E-09	1.18E-09	2.18E-07	2.53E-08	3.05E-07	2.18E-06	2.52E-06	1.75E-07	3.10E-08
	RUA	2.18E-11	2.88E-11	3.37E-11	2.23E-11	4.86E-11	3.57E-10	5.36E-11	4.46E-11	1.01E-08	3.32E-09	3.56E-08	2.86E-07	3.02E-07	2.23E-08	3.22E-09
